# Aberrant Expression of PAFAH1B3 Affects Proliferation and Apoptosis in Osteosarcoma

**DOI:** 10.3389/fonc.2021.664478

**Published:** 2021-05-31

**Authors:** Jian Fan, Yi Yang, Ji-kui Qian, Xin Zhang, Jia-qing Ji, Li Zhang, Shan-zhu Li, Feng Yuan

**Affiliations:** Department of Orthopedics, Tongji Hospital, Tongji University, Shanghai, China

**Keywords:** osteosarcoma, platelet activating factor acetylhydrolase 1B3, cell proliferation, apoptosis, therapeutic target

## Abstract

Osteosarcoma is a major malignant tumor of bone and soft tissue, which is presenting with early metastasis and a high mortality rate. Platelet activating factor acetylhydrolase 1B3 (PAFAH1B3), a cancer-relevant molecular, was found to play a vital role in tumorigenesis and aggressiveness in several cancer types. However, the roles and the regulating mechanisms of PAFAH1B3 in osteosarcoma progression remain unclear. PAFAH1B3 expression was detected by immunohistochemistry in 83 osteosarcoma tissues and 44 paired adjacent normal bone tissues. *In vitro*, loss-of-function assay was performed to explore the role of PAFAH1B3 in osteosarcoma cells. Tumor xenograft growth assay was used to verify the effect of PAFAH1B3 knockdown on osteosarcoma growth *in vivo*. Chip assay was carried out to investigate the mechanism in osteosarcoma proliferation regulated by PAFAH1B3. PAFAH1B3 was overexpressed in osteosarcoma tissues and cell lines. Moreover, PAFAH1B3 knockdown inhibited osteosarcoma cell proliferation and promoted apoptosis *in vitro*, and also suppressed osteosarcoma growth *in vivo*. Furthermore, the proliferative effect of PAFAH1B3 in osteosarcoma was related to the regulation of the expression of EIF4EBP1, MYC, PTGS2 and RPS6KB1. This study demonstrated the biological function of PAFAH1B3 on osteosarcoma proliferation. This research suggested that PAFAH1B3 could be a novel therapeutic target for osteosarcoma patients.

## Introduction

As one of the most common primary bone sarcoma, osteosarcoma is usually characterized by a high grade of metastasis (1). Surgery and chemotherapy are the mainstays of therapy. However, the clinical outcome for osteosarcoma patients has not been drastically improved with conventional therapies over the past 30 years (2). Therefore, identification of new therapeutic targets for osteosarcoma patients is urgently needed.

Platelet-activating factor acetylhydrolase 1B (PAFAH1B) complex is a heterotrimeric enzyme, which can regulate the activity of platelet-activating factor (PAF) by deacetylation (3). PAFAH1B3 is one of the catalytic subunits of PAFAH1B complex (4–7). Recently, upregulated PAFAH1B3 was shown among the most commonly metabolic enzymes across >1000 primary human tumors (8). Similarly, PAFAH1B3 was highly expressed in different cancer cell lines including melanoma, ovarian, and breast cancer (9) and was broadly dysregulated in a wide spectrum of cancer types (10). In addition, the reduction of PAFAH1B3 expression substantially impaired cellular survival, motility, aggressiveness and *in vivo* tumor xenograft growth of triple-negative breast cancer cells, which implied PAFAH1b3 is a critical metabolic driver of breast cancer (11). Furthermore, the inhibition of PAFAH1B3 could be an effective therapeutic strategy for BCR-ABL1+ BCP-ALL patients in combination with tyrosine kinase inhibitor (TKI) treatment (12). Moreover, PAFAH1B3 exerts its biological functions *via* modulating multiple signaling pathways, such as PAF signaling pathways, Wnt pathways in developing GABAergic neurons, and reelin pathways in participating lipid metabolism (13–16). Although, PAFAH1B3 plays pivotal roles in proliferation, migration and invasion of multiple cancer types, the precise biochemical functions of PAFAH1b3 and the subsequent regulating mechanisms in osteosarcoma remain poorly understood.

Here, we detected the expression patterns of PAFAH1B3 in osteosarcoma tissues and the adjacent normal bone samples. Moreover, loss-of-function assay was conducted *in vitro* to explore the biological function of PAFAH1B3 in tumorigenesis of human osteosarcoma cell lines. To summarize, PAFAH1B3 could be suggested as a promising therapeutic target for osteosarcoma patients.

## Materials and Methods

### Patients and Osteosarcoma Specimens

Eighty-three osteosarcoma tissues and 45 adjacent normal bone tissues were obtained from osteosarcoma patients who underwent surgical resection from June 2011 to June 2013 in Tongji Hospital (Shanghai, China). All the tissue samples were frozen immediately after resection and reserved in liquid nitrogen for further experiments. All the patients were provided informed consent for using their specimens. The use of tissue samples for this research was approved by the Ethics Committee of Tongji Hospital of Tongji University.

### Immunohistochemistry (IHC)

Immunohistochemical analysis was used to examine PAFAH1B3 expression in osteosarcoma tumor tissues and adjacent non-tumor samples. In brief, paraffin-embedded tissue sections (5 μm) were deparaffinized with xylene, rehydrated through a series of graded ethanol solutions and blocked with 3%H_2_O_2_ for 10 minutes. A citrate buffer (0.01M, pH 6.0) was used to retrieve antigens at 100°C. After rinsed in distilled water at room temperature for 10 minutes, the sections were then blocked by 10% fetal bovine serum for 30 minutes. Then, the slides were incubated with the primary PAFAH1B3 antibody (1:1000, Abcam) at 4°C overnight and rinsed twice in TBS, followed by incubation of secondary antibody at room temperature for 60 minutes. VULCAN FAST RED CHROMOGEN kit2 and a DAB substrate kit were used to perform chromogenic reaction. The slides were counterstained with hematoxylin. Finally, these sections were dehydrated by serially diluted alcohols and covered with coverslips. Immunostained slides were assessed for staining intensity scores and proportional scores. Final scores were calculated by multiplying the intensity scores by the proportion scores. Staining intensity was rated from 0 to 3 (0: negative, 1: weak, 2: moderate, 3: strong) and proportional score was defined by percentage of positive-staining cells (0, 0% stained; 1, 1%–25% stained; 2, 26%–50% stained; 3, 51%–75% stained; and 4, 76%-100% stained). Then, samples were divided into two groups according to the final scores (low expression: score ≤ 6; high expression: score > 6).

### Cell Lines and Cell Culture

Human osteosarcoma cell lines (U-2 OS and MNNG/HOS) were purchased from the American Type Culture Collection (ATCC; Manassas, VA, USA), and cultured in Dulbecco’s modified Eagle’s medium (DMEM; Corning, Tauranga, New Zealand) supplemented with 10% fetal bovine serum (FBS; Ausbian, Rockville, MD, USA). All cell lines were incubated in a humidified atmosphere with 5% CO_2_ at 37°C.

### Construction of Lentiviral PAFAH1B3-siRNA Vector and Infection

Small interfering RNA (siRNA) target sequence (TGGCACCATCAGCCATCAT) for PAFAH1B3 gene (NM_002573) was designed and a non-silencing siRNA sequence (TTCTCCGAACGTGTCACGT) was adopted as negative control (NC). siRNA constructs were synthesized and cloned into GV115 plasmid vector with Age I/EcoRI sites (TIANGEN, Shanghai, China) which contains the enhanced green fluorescent protein (EGFP) gene as a reporter with an internal CMV promoter. The PAFAH1B3-siRNA plasmid was then transfected into MNNG/HOS and U-2 OS cells with lentivirus according to the manufacturer’s instructions.

### RNA Extraction and Quantitative Real-Time PCR

Total RNA was extracted from the cultured cells using Trizol reagent (Pufei, shanghai, China) following the manufacturer’s protocol. Complementary DNA (cDNA) was synthesized by using Promega M-MLV kit (Promega, USA). Quantitative real-time polymerase chain reaction (qRT-PCR) was applied to detect the expression of mRNA. Glyceraldehyde-3-phosphate dehydrogenase (GAPDH) cDNA was used as an internal control for quantification. The following primers were used for qRT-PCR amplification: PAFAH1B3 expression were 5’- GAGAAGAACCGACAGGTGAAC-3’ (forward) and 5’- CGGCAAACAGGTGTGTAGC-3’ (reverse); GAPDH expression were 5’- TGACTTCAACAGCGACACCCA-3’ (forward) and 5’- CACCCTGTTGCTGTAGCCAAA-3’ (reverse). The PCR was performed under the following conditions: 95°C for 30 s, 40 cycles of 95°C for 5 s, 60°C for 30 s, and 95°C for 15 s, 60°C for 30 s, 95°C for 15 s. The relative mRNA expression levels were calculated by using the 2−ΔΔCt method.

### Western Blot

The proteins were extracted from the cells harvested 72 hours after transfection using RIPA lysis buffer (Biyuntian, Jiangsu, China). BCA Protein Assay Kit (Biyuntian, Ludwigshafen, Germany) was utilized to measure the protein concentrations. Proteins were separated by 10% SDS-PAGE gels and transferred onto a polyvinylidene difluoride (PVDF) membranes (Millipore, Billerica, MA, USA). The membrane was blocked in 5% skim milk for 1 h and then incubated with PAFAH1B3 antibody (ab170877, 1:500, Abcam, Cambridge, UK) at 4°C overnight. After 4 times of washing with Tris-buffered saline with Tween 20 (TBST), the membrane was incubated at room temperature for 1.5 h with anti-mouse secondary antibody (sc-2005, 1:2000, Santa Cruz). The bound antibodies were visualized with Pierce™ ECL Western Blotting Substrate (Thermo Fisher Scientific). The GAPDH was used as endogenous control.

### MTT Assay *in Vitro*

MTT assay was conducted to evaluate the rate of cell proliferation *in vitro*. In brief, the cells were seeded in 96-well plates at a density of 2×10^3^ cells/well and cultured for 1-5 days after transfection. Then, 20 μl of MTT reagent (Genview) was added to each well for 4 hours, followed by removing of supernatants in each well and adding of 100 μl of DMSO (Shiyi Chemical, Shanghai, China). The absorbance value was measured at 490 nm by using a microplate reader (Tecan infinite).

### Celigo Image Cytometry

The Celigo Image Cytometer was used to evaluate cell proliferation. PAFAH1B3-siRNA and non-silencing control cells were trypsinized and resuspended, and then were seeded into 96-well plates at 2000 cells/well (100 μL) and incubated overnight. Celigo Imaging Cytometer (Nexcelom) was used to measure the GFP expression for each well over consecutively 5 days. The data was collected for statistical data mapping and production of cell proliferation curves.

### Clone Formation Assay

Clone formation assay was performed to estimate the capacity of cell proliferation *in vitro*. PAFAH1B3-siRNA and control cells (400 cells/well) were planted into six-well plates. Culture medium was changed every 3 days. After 14 days of incubation, most single colonies containing more than 50 cells were seen as surviving colonies and visualized under a fluorescence microscope (Olympus). Then, cells were washed with PBS once and fixed in 4% paraformaldehyde for 30 minutes. The colonies were washed with PBS and stained with 500 μl Giemsa solution for 20 min. After washing with deionized distilled water for 3 times, the number of colonies was counted using fluorescence microscopy (Olympus) and images were captured by a digital camera under light microscopy. This assay was performed in triplicate.

### Apoptosis Analysis

Cell apoptosis was assessed by labeling with Annexin V-APC (eBioscience, San Diego, CA, United States) and analyzed by FACS. Cells were harvested with trypsin, centrifuged and resuspended in binding buffer. Next, cells (200 μl of the cell suspension solution) were stained by 10 μl Annexin V-APC, incubated in the dark at room temperature for 15 min, and subjected to flow cytometry (Millipore). All experiments were repeated three times.

### Tumor Xenograft Growth Assay *In Vivo*

Female BALB/c nude mice were 4 weeks old and purchased from Shanghai Lingchang Biotechnology Co. Ltd. All animal experiments were approved by the Ethics Committee of Tongji Hospital of Tongji University and performed following the animal care and use guidelines of the National Institutes of Health of USA. For the tumor growth assay, 5×10^6^ cells transfected with siPAFAH1B3 or siNC were resuspended in PBS medium and then injected subcutaneously into the right flank of nude mice. The tumor xenograft growth was monitored for 16 days. The tumors were measured every two days since their appearance. The tumor volume (cm^3^) was calculated by the following formula: π/6 × length × width^2^. On day 17, the animals were euthanized and the accurate tumor volume of each mouse was measured, weighed and compared between siPAFAH1B3 group and negative control.

### Chip Assay

GeneChip primeview human PathArray™ was applied to investigate the gene expression profiles of MNNG-HOS cells infected by LV-PAFAH1B3-RNAi for 72h. Following the manufacturer’s instructions, complementary DNA (cDNA) synthesis, reverse transcription of mRNA into cDNA and hybridization were performed. After that, microarray data was evaluated by several analyses including Signal Histogram, Relative Signal Box Plot, Pearson’s correlation analysis and Principal Component analysis (see **Supplementary Figures 1–4**). Differently expression genes (DEGs) were identified through Bayesian linear regression and normalized by Benjamini and Hochberg procedures (|Fold Change|>2 and *P <*0.05) (17). Moreover, all the DEGs were submitted to Ingenuity Pathway Analysis (IPA) (www.ingenuity.com).

### Statistical Analysis

All experiments were repeated three times independently and analyzed with Student t test. *P <*0.05 was taken as statistically significant.

## Results

### Overexpression of PAFAH1B3 in Osteosarcoma Tissues and Cell Lines

**Figure 1:**
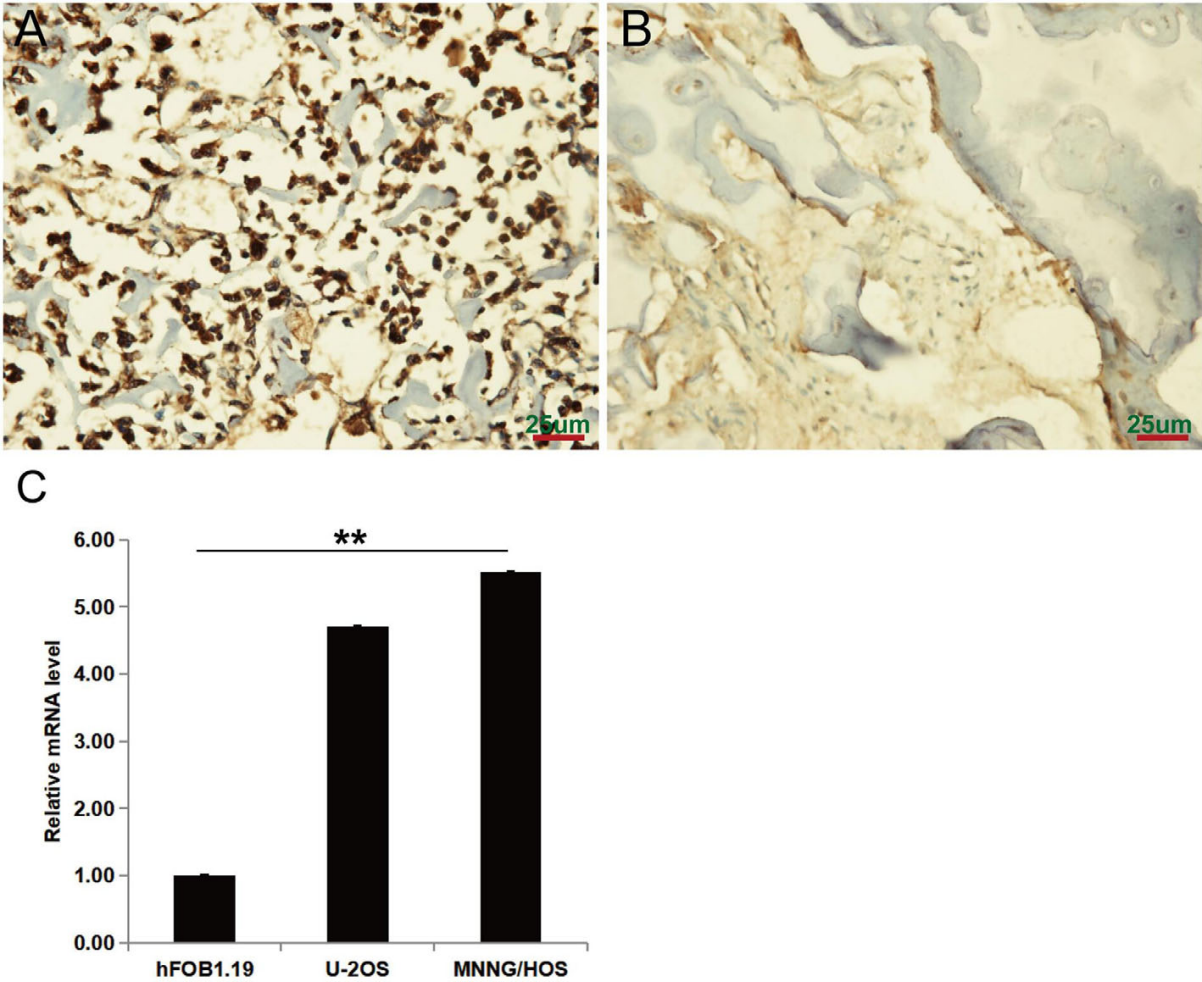
PAFAH1B3 expression in osteosarcoma tumor and adjacent non-tumor tissues. **(A)** Representative images of immunohistochemical staining for PAFAH1B3 in osteosarcoma tumor tissues, strong expression. **(B)** Representative images of immunohistochemical staining for PAFAH1B3 in adjacent non-tumor tissues: absent expression. Original magnification: 400×. **(C)** High expression of PAFAH1B3 mRNA level in U-2 OS and MNNG/HOS cell lines. Data were expressed as the mean ± s.d., **P < 0.01.

The expression level of PAFAH1B3 in osteosarcoma tumor tissues (n=83) and corresponding normal bone specimens (n=44) was detected by IHC. As shown in **Figures 1A, B**, PAFAH1B3 was significantly overexpressed in osteosarcoma tumor samples (62.7%, 52/83), but was hardly measured in the matching non-tumor tissues (22.7%, 10/44) (*P* < 0.01). In order to perform *in vitro* experiments, we selected two osteosarcoma cell lines (U-2 OS and MNNG/HOS) and analyzed corresponding PAFAH1B3 expression by using qRT-PCR. High expression of PAFAH1B3 mRNA level was confirmed in both U-2 OS and MNNG/HOS cell lines (**Figure 1C**). Therefore, U-2 OS and MNNG/HOS cell lines were selected for subsequent experiments. Overall, these findings suggest that PAFAH1B3 is overly expressed in osteosarcoma.

### PAFAH1B3 Expression Is Suppressed by Infection of Lentivirus-Mediated siRNA in Osteosarcoma Cells

**Figure 2:**
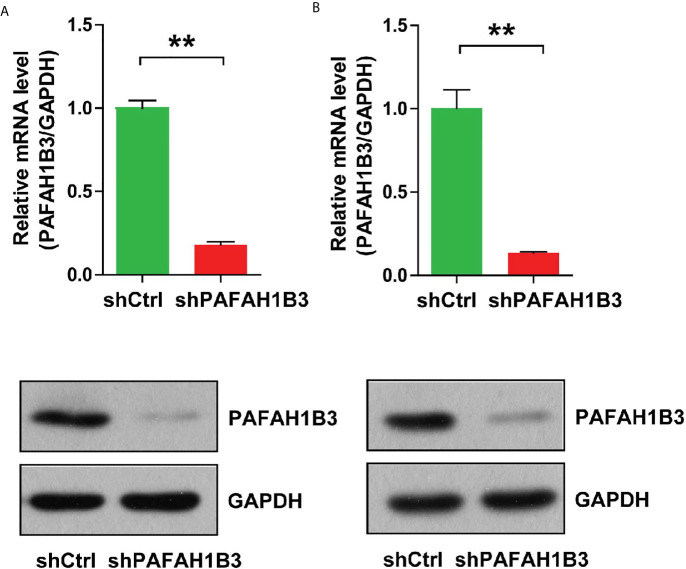
**(A)**The knockdown efficiency of PAFAH1B3 by infection of lentivirus-mediated siRNA and the negative control siRNA in MNNG/HOS cells was verified by qRT-PCR and Western blot. Data were expressed as the mean ± s.d., **P < 0.01. **(B)** The knockdown efficiency of PAFAH1B3 by infection of lentivirus-mediated siRNA and the negative control siRNA in U-2 OS cells was verified by qRT-PCR and Western blot. Data were expressed as the mean ± s.d., **P < 0.01.

Loss-of-function assay was carried out to explore the role of PAFAH1B3 in osteosarcoma cells. PAFAH1B3 knockdown was achieved by transfection with PAFAH1B3-specific siRNA plasmids. The transfection efficiency was verified by qRT-PCR analysis and Western blot, respectively. As shown in **Figure 2A**, the mRNA and protein expression levels of PAFAH1B3 were significantly reduced in MNNG/HOS cells transfected with PAFAH1B3-specific siRNA (shPAFAH1B3) in comparion wih the negative control siRNA (shCtrl), respectively (*P* < 0.01). Similar results were found in transfected U-2 OS cells (**Figure 2B**). Accordingly, lentiviral-mediated siRNA infection was an effective way to knockdown PAFAH1B3 in osteosarcoma cells.

### PAFAH1B3 Knockdown Inhibited Osteosarcoma Cell Proliferation *In Vitro*

**Figure 3:**
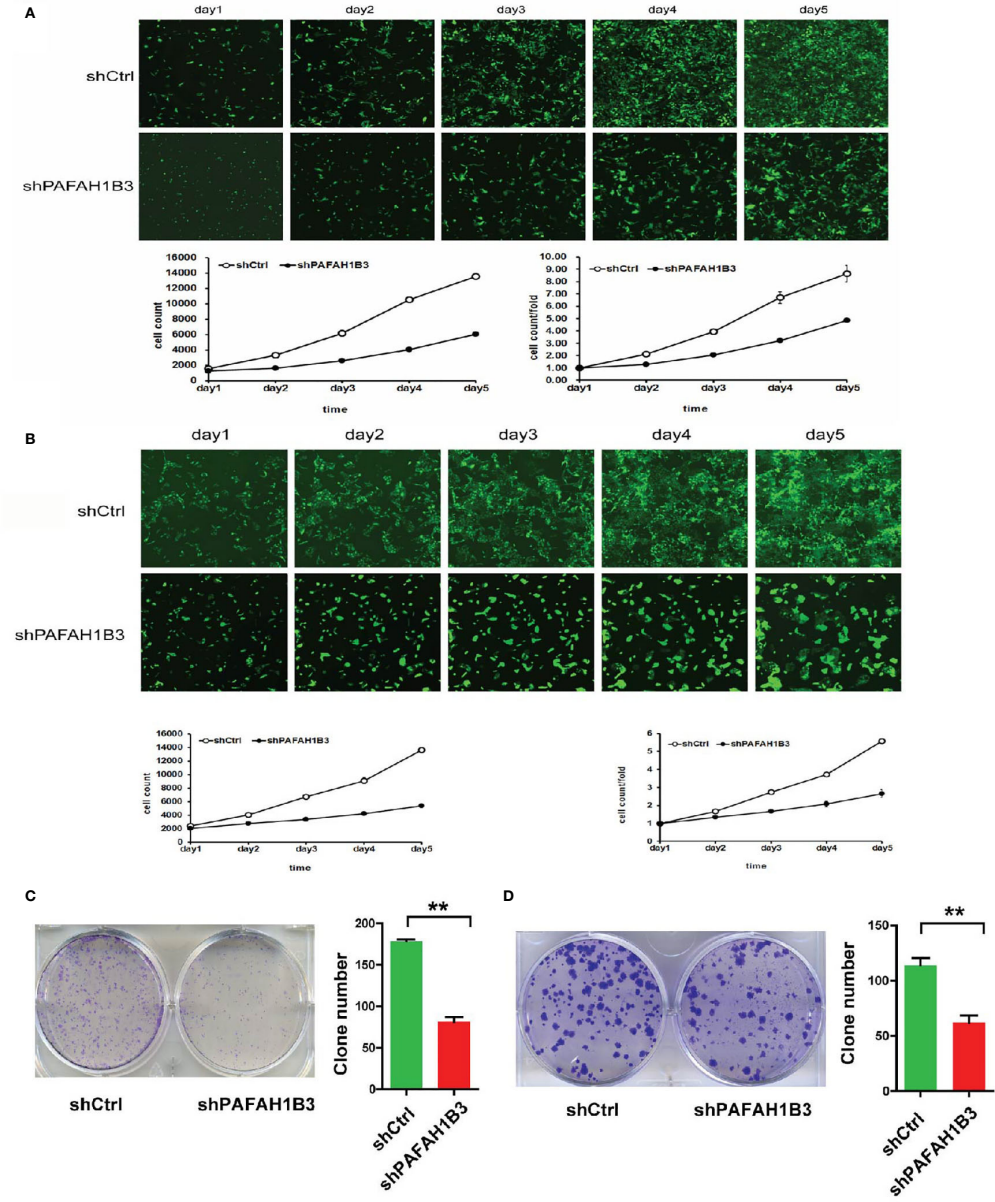
PAFAH1B3 knockdown suppressed osteosarcoma cell proliferation. **(A)** Proliferation of MNNG/HOS cells was significantly inhibited in the absence of PAFAH1B3. **(B)** Proliferation of U-2 O cells was significantly inhibited in the absence of PAFAH1B3. **(C)** The ability of forming colonies of PAFAH1B3 knockdown cells was significantly restrained compared with control cells in MNNG/HOS cell line. Data were expressed as the mean ± s.d., **P < 0.01. **(D)** The ability of forming colonies of PAFAH1B3 knockdown cells was significantly restrained compared with control cells in U-2 OS cell line. Data were expressed as the mean ± s.d., **P < 0.01.

MTT assay, Celigo image cytometry and clone formation assay were performed to investigate the effects of PAFAH1B3 knockdown on proliferation of osteosarcoma cells. Cell proliferation was analyzed using MTT assay or Celigo assay in 5 consecutive days. As depicted in **Figures 3A, B**, the cellular proliferative rate of PAFAH1B3-siRNA group was remarkably diminished in comparison with control group in both MNNG/HOS and U-2 OS cell lines. Moreover, **Figures 3C, D** showed that the ability to form colonies of PAFAH1B3 knockdown cells was dramatically restrained compared with control cells in both MNNG/HOS and U-2 OS cell lines (*P* < 0.01). Therefore, it is demonstrated that knockdown of PAFAH1B3 was associated with decreased cell proliferation and clone formation.

### PAFAH1B3 Knockdown Promoted Apoptosis in Osteosarcoma Cells *In Vitro*

**Figure 4:**
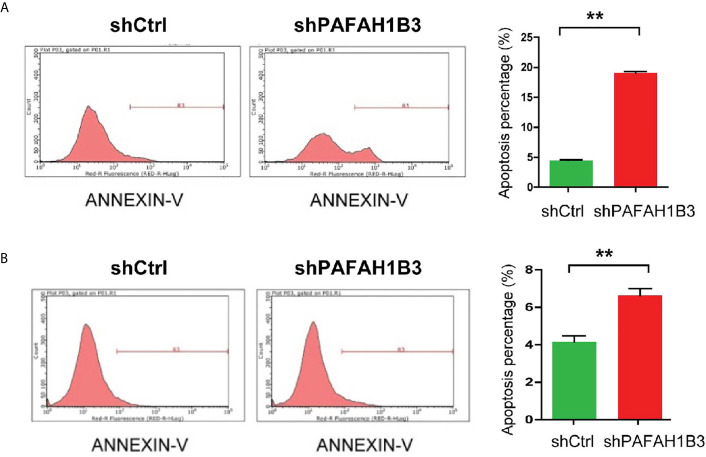
PAFAH1B3 knockdown promoted apoptosis in osteosarcoma cells. **(A)** The proportion of apoptotic cells in MNNG/HOS cell line was significantly increased in PAFAH1B3 knockdown group assessed by flow cytometry. Data were expressed as the mean ± s.d., **P < 0.01. **(A, B)** The proportion of apoptotic cells in U-2 OS cell line was significantly increased in PAFAH1B3 knockdown group assessed by flow cytometry. Data were expressed as the mean ± s.d., **P < 0.01.

To further elucidate the growth-inhibiting effect of PAFAH1B3 depletion cells, apoptosis analysis was determined subsequently by flow cytometry. The apoptotic rate assessed following Annexin V-APC staining showed that the apoptotic proportion was significantly higher in PAFAH1B3 knockdown cells than in the control cells in both MNNG/HOS and U-2 OS cell lines (**Figures 4A, B**). Therefore, these data revealed that silencing of PAFAH1B3 in osteosarcoma cells induces cell apoptosis.

### PAFAH1B3 Knockdown Suppressed Tumor Growth *In Vivo*

**Figure 5:**
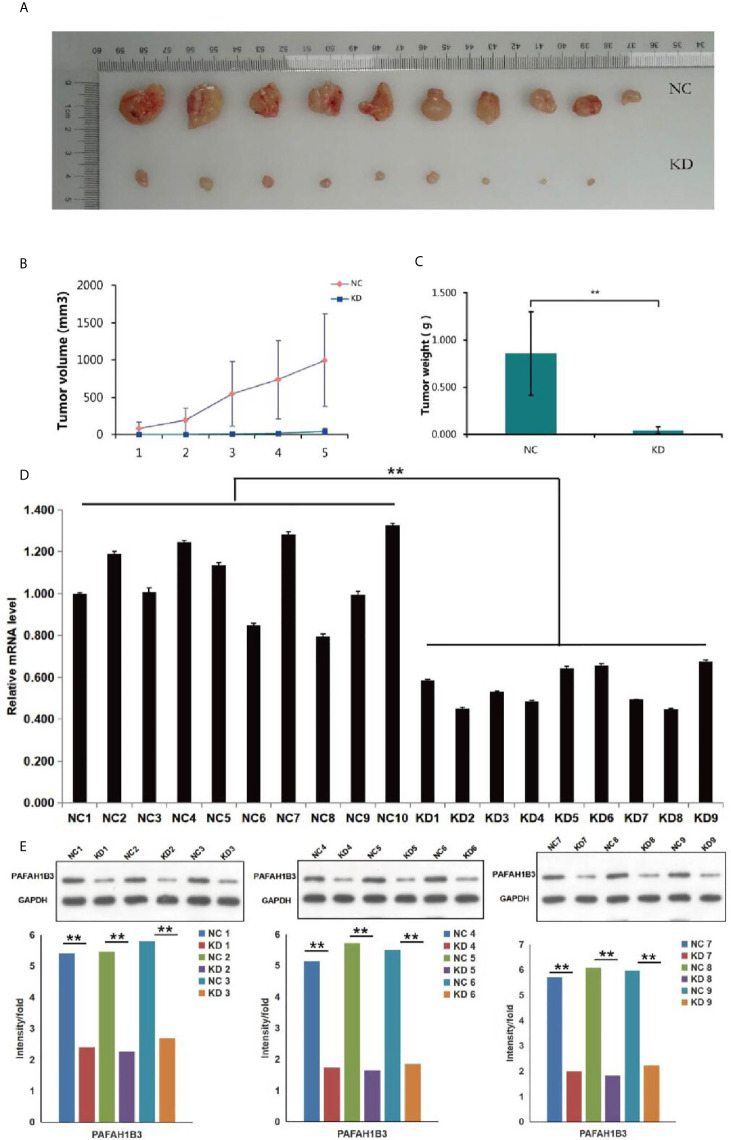
Effects of PAFAH1B3 knockdown on tumor growth *in vivo*. **(A)** The tumor volume was obviously decreased in PAFAH1B3 knockdown group. **(B)** The difference of the tumor volume was shown clearly between PAFAH1B3 knockdown group and negative control after 5 measurements. **(C)** The tumor weight was significantly declined in PAFAH1B3 knockdown group (**P < 0.01). **(D)** The expression of PAFAH1B3 mRNA level was significantly declined in PAFAH1B3 knockdown group (**P < 0.01). **(E)** The expression level of PAFAH1B3 was detected by Western blotting and significantly declined in PAFAH1B3 knockdown group (**P < 0.01). NC, negative control KD; knockdown.

To verify whether the growth-suppressing effect of PAFAH1B3 knockdown on osteosarcoma cells was related to the tumor growth *in vivo*, MNNG/HOS cells transfected with siPAFAH1B3 or siNC were subcutaneously inoculated into nude mice. The results manifested that knockdown of PAFAH1B3 distinctly decreased the tumor volume (**Figures 5A, B**) and the tumor weight (**Figure 5C**). The expression level of PAFAH1B3 mRNA and PAFAH1B3 were both significantly declined (**Figures 5D, E**). Therefore, the results suggested that silencing of PAFAH1B3 suppresses tumorigenicity of osteosarcoma.

### Bio-Informatics Analysis

**Figure 6:**
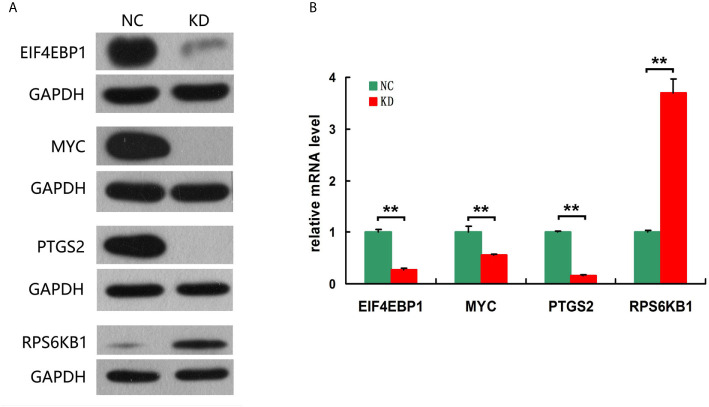
Verification the expression of 4 downstream regulatory factors related to the proliferation and apoptosis. **(A)** EIF4EBP1, MYC and PTGS2 were significantly down-regulated, while RPS6KB1 was dramatically up-regulated in protein level. **(B)** EIF4EBP1, MYC and PTGS2 were significantly down-regulated, while RPS6KB1 was dramatically up-regulated in mRNA level.

To explore the mechanism in osteosarcoma proliferation regulated by PAFAH1B3, transcriptome analysis was performed to compare the gene expression profiles in control MNNG-HOS cells and PAFAH1B3 knockdown MNNG-HOS cells. After validating the quality of GeneChip PathArray data, classic pathways analysis and disease and function analysis were performed to show cluster status of differentially expressed genes and relative downstream molecules. Among them, Osteoarthritis Pathway (**Supplementary Figure 5**) was significantly inhibited as the Z-score of this pathway was -3.202. In addition, disease and function Heat Map (**Supplementary Figure 6**) illustrates the regulation pattern of differentially expressed genes (up-regulation and down-regulation) and the status of functions and diseases (activation and inhibition). Western blot and qPCR were used to verify the expression of 30 downstream regulatory factors that related to the proliferation and apoptosis functions (see **Table 1**). Among them, we could show that EIF4EBP1, MYC and PTGS2 were significantly down-regulated, while RPS6KB1 was dramatically up-regulated in both protein and mRNA level, respectively (**Figures 6A, B**). Taken together, these data suggest that the proliferative effect of PAFAH1B3 in osteosarcoma is related to the regulation of the expression of EIF4EBP1, MYC, PTGS2 and RPS6KB1.

**Table 1:** Expression of 30 downstream regulatory factors.

Gene Symbol	Regulation	Absolute FC
EIF4EBP1	down	3.72
GSK3B	up	2.63
MYC	down	2.47
PTGS2	down	7.70
RPS6KB1	up	3.05

## Discussion

Osteosarcoma is highly metastasized and has a poor prognosis, hence it is urgent to discover novel therapeutic targets for osteosarcoma patients. Recent studies focused on identifying new metabolic drivers correlated with cancer proliferation and aggressiveness (18–20). PAFAH1B3, known as one of the most common upregulated metabolic enzymes in cancers, is important for maintaining cancer pathogenicity in breast cancer and head and neck squamous cell carcinoma (11, 20). In addition, the pharmacological inhibition or RNA interference-mediated inactivation of PAFAH1B3 could impair cancer cell survival, migration and invasiveness *in vitro* and slow tumor growth *in vivo* (10, 11, 21). However, the biological role of PAFAH1B3 in osteosarcoma has not been elucidated yet.

In this study, we identified that PAFAH1B3 was highly expressed in human osteosarcoma tumor tissues compared to normal bone tissues (**Figure 1**). Moreover, the basal expression level of PAFAH1B3 is relatively high in the selected osteosarcoma cell lines (U-2 OS and MNNG/HOS). In accordance with recent studies, PAFAH1B3 was overexpressed in breast cancers and HSCC tumor tissues, making it a promising target for cancer therapeutic intervention (11, 20). Furthermore, Gyorffy et al. showed PAFAH1B3 upregulation was associated with low recurrence-free survival in breast cancer patients overall, in lymph-node-positive tumors, and in grade 1–3 breast cancer patients (22). Moreover, overexpression of PAFAH1B3 was significantly correlated with declined overall survival and cervical lymph node metastasis in HSCC patients (20). Above all, the features of cancer-related PAFAH1B3 could be used for diagnosis and prognostic prediction of osteosarcoma. However, the association between PAFAH1B3 expression and clinical characteristics of osteosarcoma patients requires further investigation.

Subsequently, we showed that *in vitro* RNAi-mediated reduction of PAFAH1B3 resulted in substantially suppressed cell proliferation and clone formation and enhanced cell apoptosis in both MNNG/HOS and U-2 OS cells. *In vivo* experiments shown that the tumor volume and tumor weight were drastically decreased in the siPAFAH1B3 group. These results demonstrated the tumorigenic effect of PAFAH1B3 in osteosarcoma. In line with our findings, it has been reported that depletion of PAFAH1B3 inhibited cell growth and survival in breast cancer and HSCC cell lines (11, 20). Moreover, these two studies revealed that PAFAH1B3 knockdown significantly impaired cell migration and invasion potential, suggesting the pivotal role of PAFAH1B3 in keeping the aggressive property of cancer. Therefore, the invasive and metastatic effect of PAFAH1B3 in osteosarcoma remains further studies.

In the previous studies, the biological function of PAFAH1B3 was found to hydrolyze the lipid named platelet activating factor (PAF) (13, 23). However, a recent study demonstrated that PAFAH1B3 knockdown did not change PAF levels or PAFAH hydrolytic activity, indicating that the impact of PAFAH1B3-mediated cancer pathogenicity may not depend on PAF signaling pathways (11). This inhibition caused elevated levels of key tumor-suppressing lipids in lipid metabolism, including phosphatidylcholine, lysophosphatidyl choline, phosphatidyl serine, ceramide and triacylglycerol, generating anti-tumorigenic or pro-apoptotic effects (10, 11). On the contrary, Jae Won Chang et al. used P11, an inhibitor of PAFAH1b3, to block this enzyme making a dramatically decrease on the survival of plentiful mouse and human cancer cell lines cultivated under serum-starved conditions, which displayed that the protumorigenic function of PAFAH1b3 relies on its activity, rather than just expression (21). Thus, it would be our future interest to investigate the underlying mechanisms and direct substrates of PAFAH1B3 in osteosarcoma.

Ingenuity Pathway Analysis (IPA) is an analysis software (www.ingenuity.com), which establishes a visualized experimental system to understand the properties of various molecules such as genes, proteins, chemicals, and drugs and their interaction networks (24, 25). EIF4EBP1, MYC, PTGS2 and RPS6KB1 regulatory factors were testified by disease and function analysis, which were related to the proliferation and apoptosis functions. Overexpression of EIF4EBP1 has been found in various carcinomas (brain, CNS, CRC, kidney and other cancer types), and EIF4EBP1 acted as a hypoxia-inducible switch to promote tumor angiogenesis, survival in advanced cancer (26). Evidence suggested that MYC triggers some gene amplification to promote cell growth and proliferation. Deregulated MYC expression and loss of checkpoint components, such as TP53, trigger malignant transformation by MYC (27). PTGS2 induces cancer stem cell (CSC)-like activity, and promotes apoptotic resistance, proliferation, angiogenesis, inflammation, invasion, and metastasis of cancer cells (28). PTGS2 activation produces prostaglandin E2 (PGE2), which acts on a number of cell signaling pathways involving cell proliferation, angiogenesis, apoptosis, invasion, and immunosuppression which could increase tumor progression (29). In consistence with the literature, we found EIF4EBP1, MYC, PTGS2 were down-regulated in PAFAH1B3 knockdown osteosarcoma cells, resulting the deficiency in tumor growth and proliferation. Overall, this study has paved a foundation for elucidating PAFAH1B3 affects proliferation and apoptosis in osteosarcoma.

However, there are several limitations in our study. Firstly, this is a single-center study, the clinical value of PAFAH1B3 was investigated in a relatively small patient sample. Thus, large multicenter studies including large cohorts of osteosarcoma patients are warranted to study the prognosis of ostesarcoma. Secondly, in the current study, we mainly focused on the proliferative function of PAFAH1B3 in osteosarcoma, the prometastatic role of PAFAH1B3 requires further investigation. Last but not least, although we have identified the correlation between PAFAH1B3 and osteoarthritis pathway, the detailed underlying mechanism remains unclarified in this study. Therefore, we will validate these findings and expand our investigation in the future.

## Data Availability Statement

The original contributions presented in the study are included in the article/**Supplementary Material**. Further inquiries can be directed to the corresponding authors.

## Ethics Statement

The studies involving human participants were reviewed and approved by Ethics Committee of Tongji Hospital of Tongji University. The patients/participants provided their written informed consent to participate in this study.

## Author Contributions

JF, YY, and JQ: drafting and refining the manuscript. LZ, SL, and FY: critical reading of the manuscript. All authors contributed to the article and approved the submitted version.

## Funding

The present study was supported by the grants from the Shanghai Key Clinical Discipline Program (No.2017ZZ02004, to JF) and the Foundation of Shanghai Municipal Bureau of Health (No.201840026, to JF) and National Natural Science Foundation of China (No. 81802721, to YY).

## Conflict of Interest

The authors declare that the research was conducted in the absence of any commercial or financial relationships that could be construed as a potential conflict of interest.
