# Supplementary figures and images for: Aberrant Expression of PAFAH1B3 Affects Proliferation and Apoptosis in Osteosarcoma
Source: Front Oncol. 2021 May 31;11:664478. doi: 10.3389/fonc.2021.664478 (PMC8201501; doi:10.3389/fonc.2021.664478)

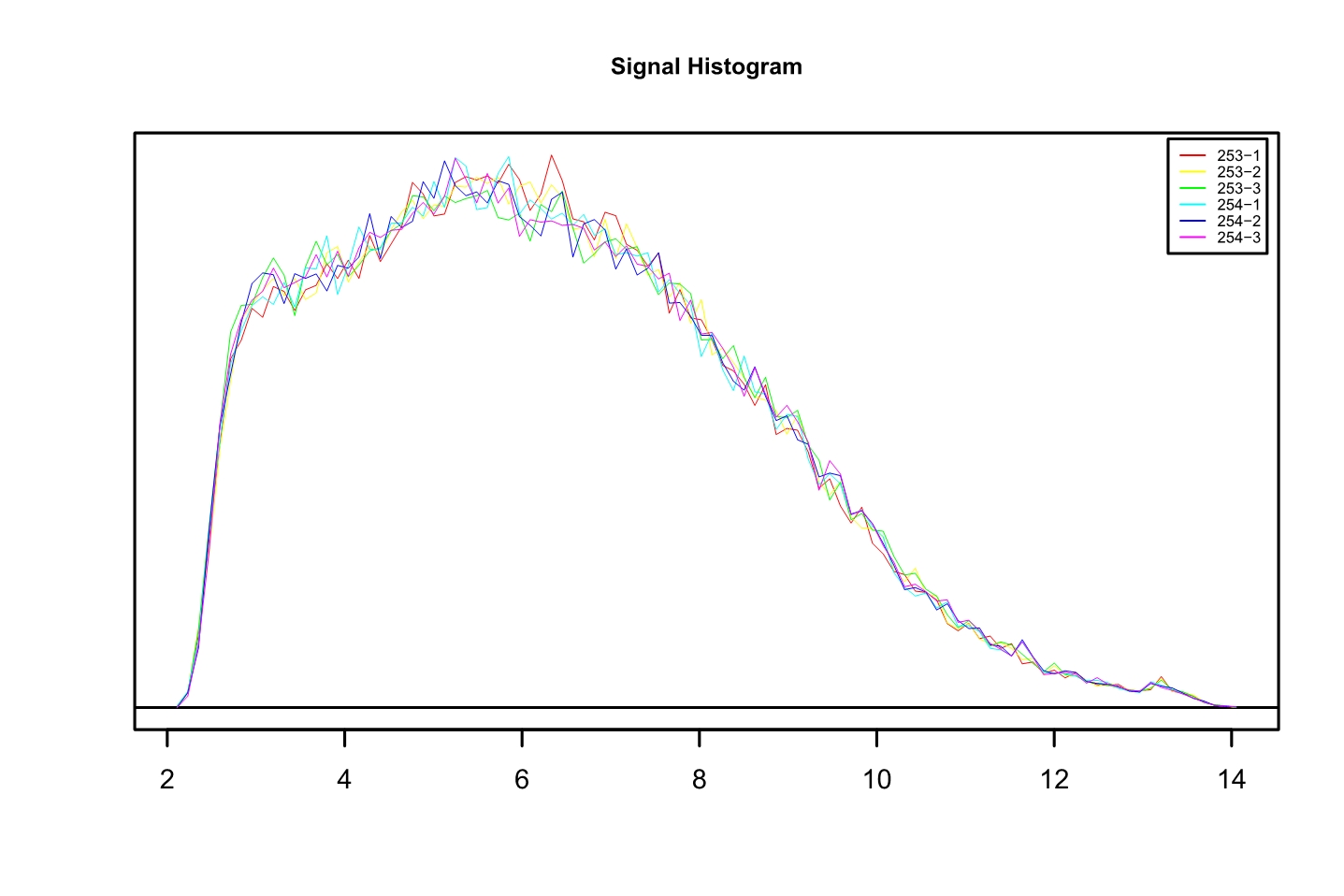

Supplement: Supplementary file 1 [file Image_1.jpeg]

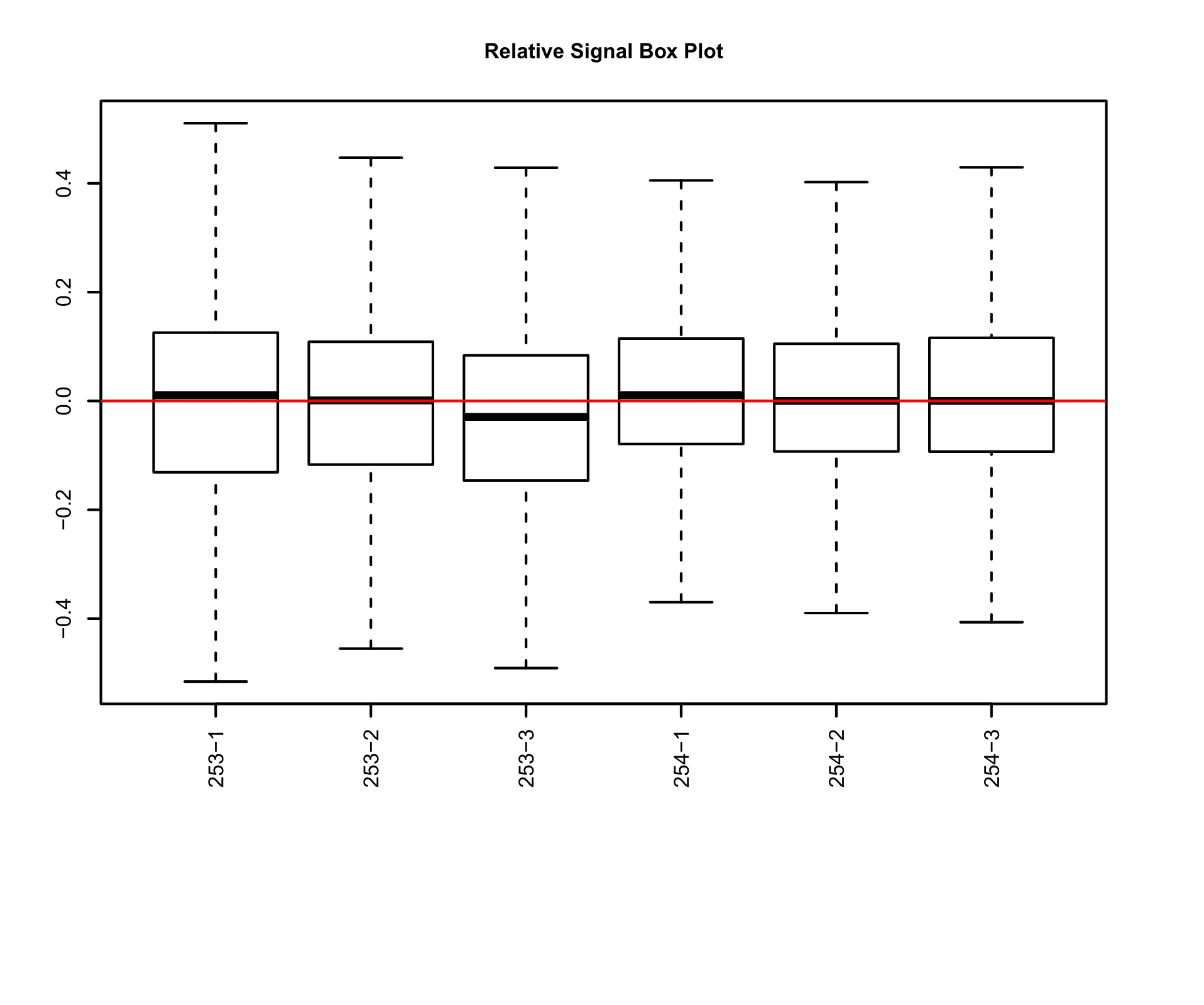

Supplement: Supplementary file 2 [file Image_2.jpeg]

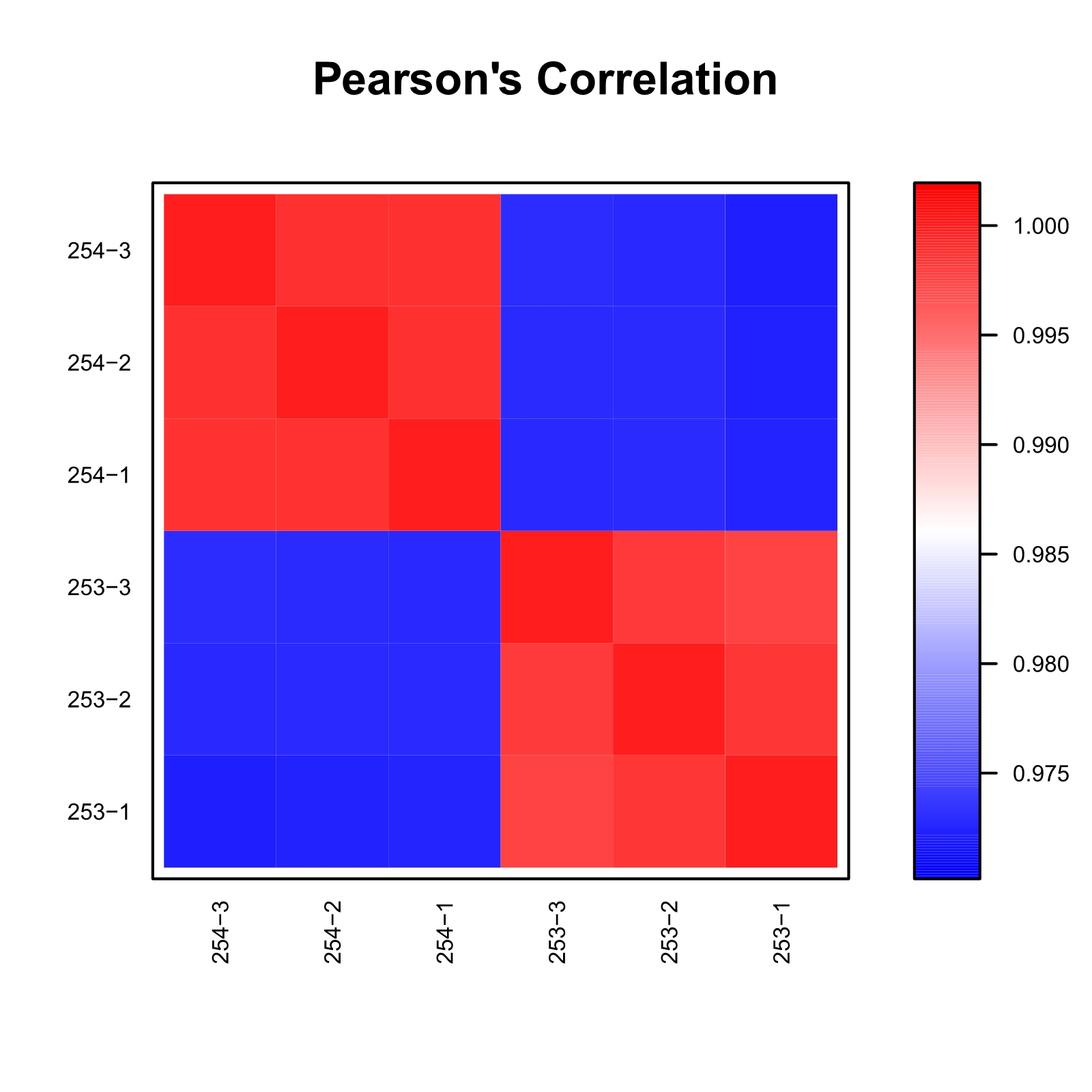

Supplement: Supplementary file 3 [file Image_3.jpeg]

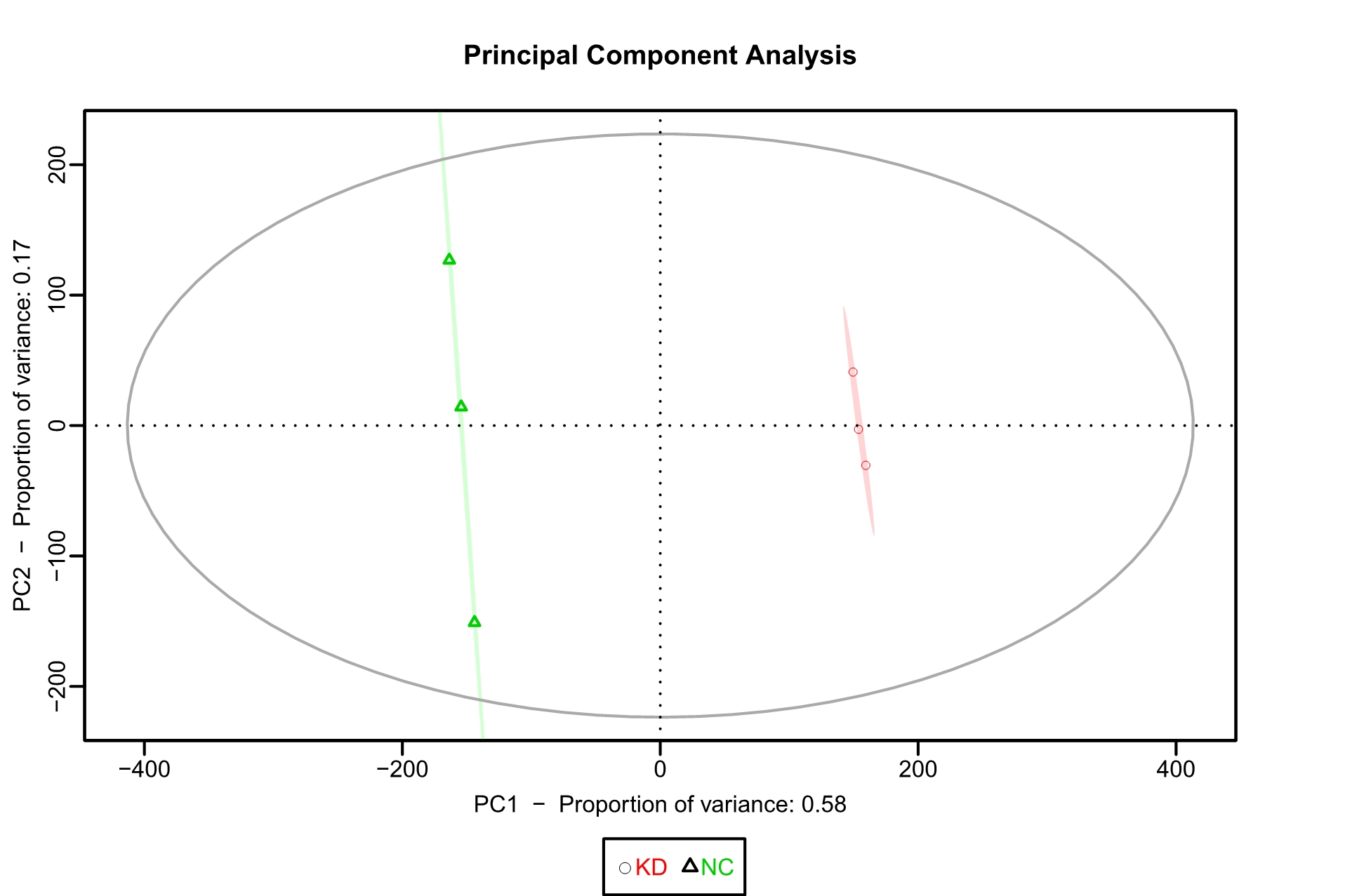

Supplement: Supplementary file 4 [file Image_4.jpeg]

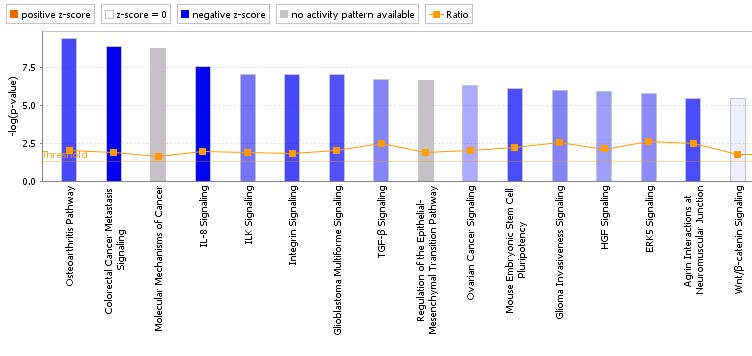

Supplement: Supplementary file 5 [file Image_5.jpg]

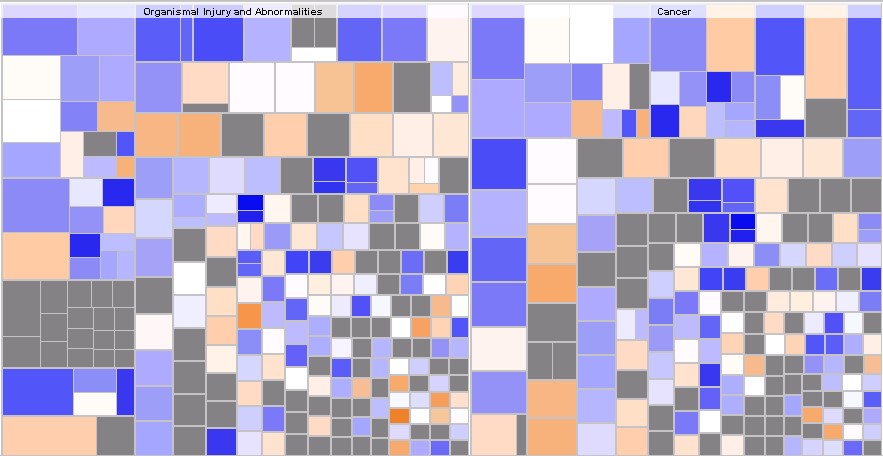

Supplement: Supplementary file 6 [file Image_6.jpg]
